# On the placement of the Cretaceous orthopteran *Brauckmannia groeningae* from Brazil, with notes on the relationships of Schizodactylidae (Orthoptera, Ensifera)

**DOI:** 10.3897/zookeys.77.769

**Published:** 2011-01-26

**Authors:** Sam W. Heads, Léa Leuzinger

**Affiliations:** 1 Illinois Natural History Survey, Institute of Natural Resource Sustainability, University of Illinois at Urbana-Champaign, Illinois, USA; 2 Department of Geosciences, University of Fribourg, Switzerland

**Keywords:** Orthoptera, Ensifera, Gryllidea, Schizodactylidae, Brauckmanniidae, *Brauckmannia*, *Schizodactylus*, phylogeny, Crato Formation, Early Cretaceous, Brazil

## Abstract

The fossil orthopteran Brauckmannia groeningae Martins-Neto (Orthoptera, Ensifera) from the Early Cretaceous Crato Formation of Brazil, currently misplaced at both the genus and family level, is transferred to the family Schizodactylidae and assigned to the extant genus Schizodactylus Brullé; ergo, Brauckmannia enters synonymy under Schizodactylus and Brauckmanniidae enters synonymy under Schizodactylidae. Schizodactylus groeningae (Martins-Neto), **comb. n.** agrees in size and general habitus with extant members of the genus, but can be readily separated by the robust, subovoid form of the metatibiae and the distinctive morphology of the lateral metabasitarsal processes. This species represents the first fossil occurrence of Schizodactylidae and the only New World record of this ancient lineage. Phylogenetic relationships of the schizodactylids are reviewed and a sister-group relationship with Grylloidea advocated based on a reappraisal of morphological and molecular evidence.

## Introduction

The Lower Cretaceous Crato Formation of northeastern Brazil is famous for the truly exquisite preservation of its remarkable fossil assemblage (e.g. Grimaldi and Engel 2005; Heads et al. 2005, 2008; Martill et al. 2007 and contributions therein). Of the many animal and plant taxa hitherto reported from the Crato palaeobiota, insects are without doubt the most diverse and account for more than 75 percent of the total number of species described from the formation to date. Insect fossils occur in superabundance within the laminated limestones of the Nova Olinda Member, where they are frequently preserved as complete, fully articulated individuals with wings, setae, soft tissues and even original colour patterns (Grimaldi 1990; Martill and Frey 1995; Heads et al. 2005; Martill et al. 2007). More than 300 species representing over 20 insect orders have been described from the Crato Formation to date (Martill et al. 2007; Heads et al. 2008) and given that the most diverse groups (notably Coleoptera, Hymenoptera, Diptera and Hemiptera) remain almost entirely unstudied, the total number of species is certain to be much higher. Indeed, this remarkable deposit is perhaps the most important source of fossil insects yet encountered in the Cretaceous of Gondwana (Heads et al. 2008), with the potential to shed light on the evolution and biogeography of the insects during what is arguably one of the most dynamic periods in their history.

Orthoptera (crickets, katydids, grasshoppers and their kin) are well-represented in the Crato Formation (Heads and Martins-Neto 2007) and constitute the most frequently encountered insect fossils in the Nova Olinda laminites. Both orthopteran suborders are present, including a diverse assemblage of Ensifera comprising the superfamilies Grylloidea, “Hagloidea” and Stenopelmatoidea *sensu lato* (Martins-Neto 1991, 2007; Heads and Martins-Neto 2007). Caelifera are represented by primitive Tridactyloidea, Eumastacoidea and stem-acridomorphs of the paraphyletic ‘locustopsoid’ complex (Martins-Neto 2003; Heads 2008). Crickets (Grylloidea) are perhaps the most diverse component of this assemblage, though the Elcanidae are by far the most abundant (Heads and Martins-Neto 2007). Fossil Orthoptera are rare in most Mesozoic deposits, though in the Crato Formation they represent as many as 30 per cent of all fossil insect specimens (Bechly 1998; Martill et al. 2007; Heads et al. 2008). This remarkable diversity and abundance highlight the importance of the assemblage and yet, despite over 20 years of research, a comprehensive treatment of the Orthoptera is still unavailable. Nevertheless, a detailed taxonomic revision is now underway (Heads in prep.) and will undoubtedly shed much-needed light not only on the evolution and biogeography of Cretaceous Orthoptera, but also on the origins of the modern fauna.

The unusual orthopteran Brauckmannia groeningae was described by Martins-Neto (2007: 3–4, fig. 1) based on a single specimen from a large quarry complex to the northeast of Mina Pedra Branca, 4–5 km west of Nova Olinda on the northern flanks of the Chapada do Araripe (see Martill et al. 2007 for locality details). Martins-Neto (2007) assigned the genus to its own family, Brauckmanniidae, which he assigned to the superfamily Stenopelmatoidea *sensu lato*; a group comprising the Anostostomatidae (king crickets and wetas), Cooloolidae (Cooloola monsters), Gryllacrididae (leaf-rolling or raspy crickets) and Stenopelmatidae (Jerusalem crickets). However, Martins-Neto’s original description (2007) is cursory at best and suffers from numerous errors in the identification and interpretation of morphological structures critical to the proper placement of the fossil. Here, we present a detailed redescription of Brauckmannia groeningae based on a new and near-complete specimen, allowing us to establish its true identity as a species of the extant genus Schizodactylus Brullé, 1835 and thus, the first fossil representative of the Schizodactylidae, or splay-footed crickets.

## Material and methods

The holotype of Brauckmannia groeningae is in the private collection of Rafael Gioia Martins-Neto, referred to in his publications as the ‘Coleção de Sociedad Brasileira de Paleoartropodologia’ with the number RGMN 500. This collection was apparently stored at his home in Ribeirão Preto, São Paulo, though since his death in August 2010 its whereabouts are unknown. As a result, any discussion of the holotype presented herein is based entirely on the illustrations in the original description (Martins-Neto 2007). However, we were recently able to examine a second, more completely preserved specimen in the Museum für Naturkunde, Berlin, Germany (MfNB). This new specimen is evidently conspecific with the holotype and allows us to redescribe the species and more accurately determine its systematic placement.

The MfNB specimen was studied using a Zeiss Stemi SV11 zoom stereomicroscope and drawings produced with the aid of a camera lucida. Macro photographs were taken using a Nikon D700 digital SLR with a 60 mm macro objective. In addition, the following extant material was also examined: Schizodactylus brevinotus Ingrisch, 2002; Schizodactylus inexpectatus (Werner, 1901); Schizodactylus burmanus Uvarov, 1935; Schizodactylus monstrosus (Drury, 1773); Schizodactylus hesperus Bei-Bienko, 1967; Comicus arenarius Ramme, 1931; Comicus calcaris Irish, 1986; Comicus campestris Irish, 1986; and Comicus capensis Brunner von Wattenwyl, 1888. Terminology used follows that normally employed for Orthoptera, with the following distinctions concerning tibial armature: ‘spine’ refers to elongate, distally pointed, unsocketed processes; and ‘spur’ refers to all socketed processes of variable form (e.g. spine-like, blade-like, lobate, etc.). The geology, stratigraphy, environment and palaeobiota of the Crato Formation were most recently reviewed by Martill et al. (2007) and contributions therein.

## Systematics

### Family Schizodactylidae

Blanchard, 1845

Schizodactylites Blanchard 1845: 249.
Schizodactylinae Ramme 1931: 163.
Schizodactylidae Ander 1939: 622.
Brauckmanniidae Martins-Neto 2007: 3, syn. n.

#### Comments.

The Schizodactylidae, or splay-footed crickets, are a relict group of primitive Ensifera notable for their uniquely modified tarsi, which bear distinctive lobe-like lateral processes that serve to support the insects as they walk around their sandy habitats. The family is traditionally subdivided into two monotypic subfamilies: Schizodactylinae Blanchard, 1845 containing the type genus Schizodactylus Brullé, 1835 found primarily in India, Pakistan, Afghanistan and parts of Southeast Asia; and Comicinae Ander, 1939 containing the apparently paedomorphic genus Comicus Brunner von Wattenwyl, 1888 found only in southern parts of Africa. Schizodactylids are primarily nocturnal and are thought to be active predators (Fletcher 1914). Indeed, species of Schizodactylus have an intimidating habitus and clear predatory adaptations including raptorial prothoracic legs and powerful, enlarged mouthparts. Sub-social behaviour and cannibalism have been observed in populations of Schizodactylus monstrosus (Drury, 1773) (Choudhuri and Bagh 1974) and Schizodactylus inexpectatus (Werner, 1901) was recently the subject of a detailed biological and ecological study by Aydin and Khomutov (2008).

### Genus Schizodactylus

Brullé, 1835

Schizodactylus Brullé 1835: 161. Type species: Gryllus monstrosus Drury, 1773.
Schizocephalus Brunner von Wattenwyl 1888: 313, lapsus calami.
Dactylocomicus Karny 1931: 102. Type species: Comicus inexpectatus Werner, 1901.
Brauckmannia Martins-Neto 2007: 4. Type species: Brauckmannia groeningae Martins-Neto, 2007, syn. n.

#### Diagnosis.

The genus Schizodactylus as presently defined comprises all large and robust schizodactylids with wings developed and, with the exception of Schizodactylus inexpectatus in which the wings are reduced, extending beyond the apex of the abdomen where they terminate in a conspicuous coil. Schizodactylus species are further characterised by greatly enlarged mouthparts, a broad diamond-shaped labrum, and strong laterally compressed pro- and mesothoracic legs. The genus is readily separated from Comicus, the only other genus in the family, which is characterised by a markedly smaller and more gracile body (usually less than 25 mm in length), long, slender legs, and complete reduction of the wings.

#### Comments.

Given the presence of distinctively coiled wings and well-developed lateral processes on the tarsi, there can be no doubt as to the placement of Brauckmannia groeningae in Schizodactylidae. In addition, there are no characters preserved in either the holotype (so far as can be seen from Martins-Neto’s illustrations) or the new material described below to exclude the species from the genus Schizodactylus. Both fossil specimens match closely the general habitus, tarsal morphology and metatibial armature of extant Schizodactylus species, and there are no convincing apomorphies to support separate generic placement.

### Schizodactylus groeningae

(Martins-Neto, 2007) comb. n.

Figs 1–2

Brauckmannia groeningae Martins-Neto 2007: 4, fig 1.

#### Material examined.

Near-complete adult (sex indet.), MfNB-I.2079. Brazil, Ceará, Chapada do Araripe; Crato Formation, Nova Olinda Member, Lower Cretaceous.

#### Diagnosis.

Schizodactylus groeningae is distinguished from all congeners by the following characters: [1] robust and acutely subovoid metatibiae (in all extant species the metatibiae are of equal width along their entire length); and [2] distinctive blade- or paddle-like lateral processes arising in the distal half of the metabasitarsus (in all extant species these processes are triangular with a broad base and acute, posteriorly directed apex, and arise within the proximal half of the metabasitarsus).

#### Description of MfNB-I.2079.

**Figures 1–2.**
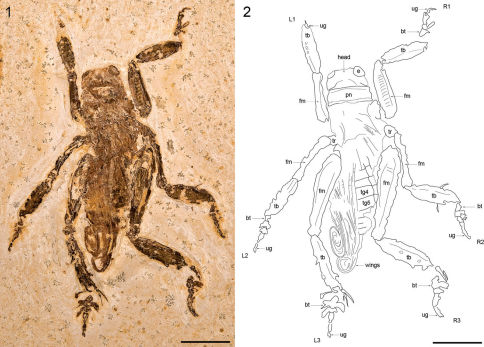
Schizodactylus groeningae (Martins-Neto, 2007), comb. n. from the Lower Cretaceous Crato Formation of Brazil. **1** Photograph of MfNB-I.2079 **2** Camera lucida drawing of MfNB-I.2079. Abbreviations: **R1** – right prothoracic leg; **L1** – left prothoracic leg; **R2** – right mesothoracic leg; **L2** – left mesothoracic leg; **R3** – right metathoracic leg; **L3** – left metathoracic leg; **bt** – basitarsus; **fm** – femur; **pn** – pronotum; **tb** – tibia; **tr** – trochanter; **ug** – ungues. Scale bars represent 10 mm.

Large, near-complete specimen preserved in slightly oblique, dorsal aspect (Figs 1, 2). Head capsule robust, 8.46 mm wide at genae; vertex short, c. 2.43 mm from occipital margin as preserved; occipital foramen large, broad; interocular distance 3.98 mm; compound eyes large, c. 2.61 mm wide dorsally. Pronotum markedly wider than long with distinctive marginal sulci; medial length 3.41 mm; width 9.36 mm. Pterothorax poorly preserved, c. 9.20 mm long. Wings incompletely preserved basally, extending posteriorly beyond abdominal apex, tightly folded in a distinctive apical coil. Abdomen somewhat crushed dorsolaterally, c. 16.12 mm long as preserved (apical part missing); first tergite (tg1) largely indistinct, at least 1.45 mm long; tg2 1.96 mm long; tg3 1.83 mm long; tg4 2.17 mm long; tg5 2.35 mm long; remaining tergites incompletely preserved but shorter than previous tergites; right lateral parts of abdominal sternites 1 through 5 visible next to corresponding tergal sclerites; pleural margin distinct. Total body length measured from fastigium verticis to abdominal apex 34.91 mm. Profemora robust and laterally compressed, 12.33 mm long; left profemur preserved in dorsal aspect, with distinct longitudinal dorsal carina; right profemur preserved in lateral aspect, with prominent transverse dorsolateral striae and distinct longitudinal inferior carina. Protibiae robust and lateral compressed, markedly inflated and acutely subovoid in form, 10.24 mm long; left protibia incompletely preserved in oblique dorsal aspect, bearing at least three spur sockets on outer lateral margin, spurs themselves not preserved; right protibia preserved in lateral aspect and somewhat crushed. Left prothoracic leg (L1 in Fig. 2) with distal part missing, only apical part of ungues preserved. Right prothoracic leg (R1 in Fig. 2) with tarsus incompletely preserved, at least 6.52 mm in total length; basitarsus subcylindrical in form, inflated apically, at least 1.75 mm long; second tarsomere short, c. 1.21 mm long, with prominent blade-like lateral process, 2.35 mm long; third tarsomere 2.10 mm long, with stout lateral process, 0.95 mm long; fourth tarsomere indistinctly preserved; ungues incompletely preserved, strongly curved, 1.44 mm long. Mesotrochantora small, c. 2.40 mm long and c. 2.20 mm wide. Mesofemora very slender, somewhat curved, inflated slightly at both the base and geniculae, 11.26 mm long and 1.46 mm wide at midlength. Mesotibiae similar in form to protibiae but somewhat larger and more acutely subovoid in lateral aspect, 10.75 mm long; left mesotibia incompletely preserved in dorsal aspect; right mesotibia preserved in lateral aspect, 3.36 mm wide at midlength, with two short subapical spurs preserved. Left mesothoracic leg (L2 in Fig. 2) with tarsus preserved in dorsal aspect, 8.17 mm long; basitarsus small, 1.15 mm long; second tarsomere 1.25 mm long with broad, incomplete lateral processes at least 1.49 mm long; third tarsomere 1.28 mm long with stout lateral processes at least 0.62 mm long; fourth tarsomere basally inflated, apically slender, 2.18 mm long; ungues at least 2.08 mm long. Right mesothoracic leg (R2 in Fig. 2) with tarsus preserved in lateral aspect, c. 8.10 mm long; basitarsus small, 1.55 mm long; second tarsomere 1.15 mm long with broad, apically incomplete lateral process at least 1.58 mm long; third tarsomere 1.23 mm long with incompletely preserved lateral process at least 0.95 mm long; margin between fourth tarsomere and ungues indistinct, combined length c. 3.91 mm. Metafemora large, robust, 19.78 mm long. Metatibiae markedly shorter than metafemora, 12.58 mm long; acutely subovoid in form, though more elongate than pro- and mesotibiae. Left metatibia preserved in dorsal aspect, somewhat crushed, with poorly preserved spines along dorsolateral margins; first apical spur 3.62 mm long; second apical spur 3.07 mm long; third apical spur incompletely preserved and visible only as a thin, 0.86 mm long fragment immediately adjacent and inferior to the second apical spur; sixth apical spur partially preserved in lateral aspect, 2.88 mm long and 0.96 mm wide apically. Right metatibia preserved in lateral aspect, 3.29 mm wide at midlength, with bases of poorly preserved spines visible along the dorsal margin; first apical spur blade-like, 3.80 mm long; second apical spur incompletely preserved immediately adjacent and inferior to the first apical spur, 2.06 mm long. Left metathoracic leg (L3 in Fig. 2) with tarsus preserved in dorsal aspect, at least 8.76 mm long; basitarsus well-developed, at least 2.80 mm long, with large, broadly paddle-like lateral processes at least 2.41 mm long and 1.78 mm wide; second tarsomere short, 0.98 mm long, with large, blade or paddle-like lateral processes at least 2.28 mm long and 0.94 mm wide; third tarsomere incomplete, at least 1.98 mm long; combined length of fourth tarsomere and ungues c. 2.98 mm. Right metathoracic leg (R3 in Fig. 2) with tarsus preserved in lateral aspect, at least 9.50 mm long; basitarsus well-developed, at least 2.66 mm long, with large, paddle-like lateral process at least 2.37 mm long; second tarsomere short, 1.04 mm long, incompletely preserved; third tarsomere indistinct, with basal part of small lateral process visible; fourth tarsomere and ungues poorly preserved, combined length 3.97 mm.

#### Comments.

The photograph of the holotype provided by Martins-Neto (2007: fig. 1A) is of rather poor quality, though it is obvious that the specimen is not as well preserved as MfNB-I.2079. Moreover, the accompanying drawing (fig. 1B) is not only incomplete (for reasons that are unclear, the drawing only depicts part of the specimen) but does not correspond entirely with features clearly visible in the photograph. Nevertheless, the photograph shows sufficient details for the identification of the holotype as a schizodactylid; namely the presence of distinctive paddle-like lateral processes on the tarsi (clearly visible on both metatarsi though misidentified as ‘well-developed pulvilli’ by Martins-Neto), and apically coiled wings (not mentioned in the original description). Moreover, the holotype is clearly conspecific with MfNB-I.2079, agreeing with it not only in the relative proportions of the legs and overall size and habitus, but also in the distal origin of the metabasitarsal processes.

Schizodactylus groeningae represents the only fossil record of Schizodactylidae and confirms the antiquity of an extant lineage hitherto unknown from the fossil record. Especially significant is that the species belongs to an extant, albeit relict genus, suggesting that the initial radiation of the Schizodactylidae occurred at least during the Jurassic if not earlier. Moreover, the presence of Schizodactylus groeningae represents the only record of Schizodactylidae from the New World, confirming presence of the family in the Atlantic rift zone of South America prior to its complete separation from Africa. An arid or semi-arid local environment for the Crato hinterland was first suggested by Martill (1993) and later supported by the discovery of solifuges (Selden and Shear 1996) and diplurid spiders (Selden et al. 2006). The development of thick and laterally extensive sandstones within the Crato Formation (Heimhofer and Martill 2007) provides direct evidence for the existence of large local sand bodies and the preserved root balls of fossil plants often consist largely of sand-rich palaeosols (Mohr et al. 2007). Evidence for seasonal flash flooding is also well attested (see Martill et al. 2007 and contributions therein) and might explain how the terrestrial elements of the biota were transported into the Crato lagoon. The presence of Schizodactylus in sandy, xeric monsoonal environments today is therefore, entirely consistent with the hypothesised palaeoenvironment and would suggest that the habitat preferences of schizodactylids have changed little in over 100 million years.

## Discussion

**Figure 3.**
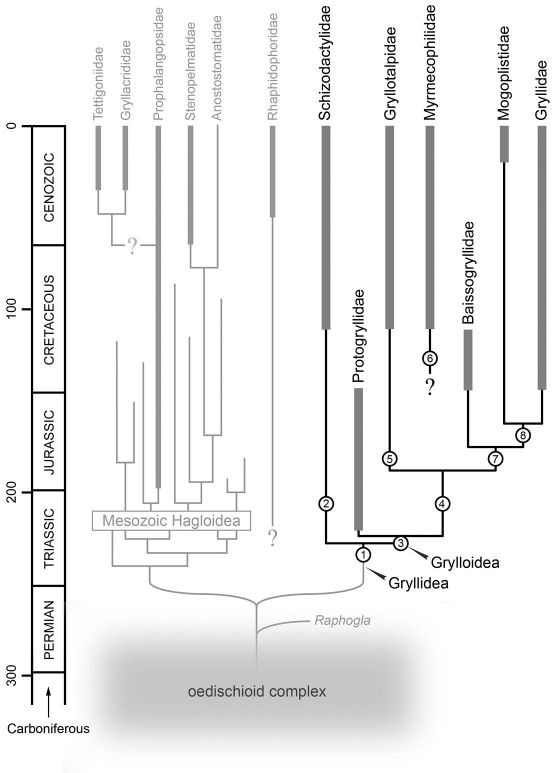
Competing hypotheses of ensiferan relationships, after: a – Ander (1939); b – Zeuner (1939); c – Ragge (1955); d – Sharov (1968); e – Gorochov (1995a, b); f– Gwynne (1995); g– Desutter-Grandcolas (2003); h– Jost and Shaw (2006); i – Legendre et al. (2010).

The relationships of the splay-footed crickets have proven somewhat controversial (see Fig. 3), with the group generally regarded as a subfamily within Gryllacrididae (Ramme 1931; Karny 1937; Zeuner 1939; Gorochov 1995a, b) or as a subfamily close to Gryllacridinae but within Stenopelmatidae (Gorochov 2001). Sharov (1968: 71) considered the group a ‘*reliktovyǐ oskolok*’ or ‘relictual fragment’ of Hagloidea, closest to Prophalangopsidae amongst the extant Ensifera. In contrast, Ragge (1955) and Gwynne (1995) considered the Schizodactylidae as occupying a basal position within Gryllidea as sister-group to the Grylloidea. Ander (1939) included the family in his ‘Tettigonioidea’ (= Tettigoniidea), basal to a lineage giving rise to the Gryllacrididae, Stenopelmatidae *sensu lato*, Prophalangopsidae and Tettigoniidae. This view was upheld at least in part by Desutter-Grandcolas (2003) who, in her landmark cladistic study of ensiferan relationships, recovered Schizodactylidae as sister-group to a clade comprising Cooloolidae, Stenopelmatidae, Anostostomatidae, Prophalangopsidae and Tettigoniidae (Fig. 3g). However, recent molecular analyses (Jost 2002; Jost and Shaw 2006; Legendre et al. 2010) have consistently failed to support such a relationship, instead recovering Schizodactylidae (represented in all three studies only by Comicus) as sister-group to the Grylloidea as proposed by Ragge (1955) and Gwynne (1995).

Morphological support for a schizodactylid–grylloid relationship comes primarily from the morphology and venation of the wings, namely: [1] marked reduction or loss of costal veins; [2] development in the tegmina of a longitudinal fold; [3] concurrent development of a distal medial fan in the tegmina; [4] development of fan-like folding in the cubital and medial systems of the hind wings; and [5] hind wing CuA two-branched. In addition, Gwynne (1995; following Ander 1939) noted the fusion of abdominal ganglion 7 with the posterior ganglionic mass (comprising fused abdominal ganglia 8 through 10) in both Schizodactylidae and Grylloidea as a potential autapomorphy of Gryllidea *sensu* Ragge (1955). Such an arrangement is also known in certain Rhaphidophoridae (e.g. Dolichopoda, Hadenoecus and all Rhaphidophorini) though in other members of the family the 7th ganglion is free (e.g. Ceuthophilus, Neonetus and Pristoceuthophilus). Indeed, the Rhaphidophoridae have traditionally been considered transitional between Grylloidea and the other Ensifera (e.g. Snodgrass 1937; Ander 1939; see also Desutter-Grandcolas 2003). The optimal tree recovered by Legendre et al. (2010) in their reanalysis of the Jost and Shaw (2006) molecular data set, placed the rhaphidophorids as sister-group to the rest of Ensifera (Fig. 3i). However, both Desutter-Grandcolas (2003) and Legendre et al. (2010) noted that none of the existing morphological or molecular datasets are adequate to test rhaphidophorid monophyly and the group could be paraphyletic with respect to Gryllidea (Schizodactylidae + Grylloidea), Tettigoniidea (the so-called ‘katydid clade’ comprising Tettigoniidae and the various ‘stenopelmatoid’ families), or both (*contra* Béthoux and Nel 2002). Clearly, comprehensive and integrated morphological-molecular and neontological-palaeontological analyses are necessary and will undoubtedly shed much-needed light on ensiferan phylogeny.

**Figure 4.**
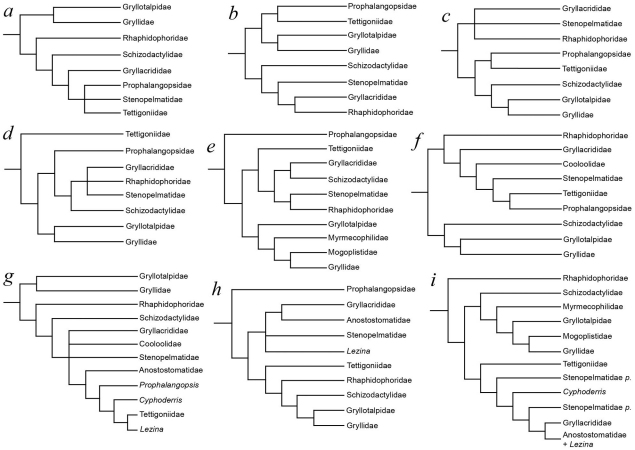
Possible relationships among major ensiferan groups (both fossil and extant), with an emphasis on the infraorder Gryllidea. Thick lines indicate known geological ranges whilst thinner lines project likely ranges based on sister-group relationships. Arabic numerals at nodes indicate autapomorphic character transformations as follows: **1** (i) reduction or loss of cubitus; (ii) development of longitudinal radio-medial fold in tegmina; (iii) development of a distal medial fan in tegmina; (iv) development of fan-like folding in cubital and medial systems of hind wings; (v) hind wing CuA two-branched; (vi) fusion of abdominal ganglion 7 with posterior ganglionic mass **2** (i) hind wings, when developed, tightly folded at rest and apically coiled in a distinctive ring; (ii) well-developed, blade or paddle-like lateral processes present on the 2nd and 3rd tarsomeres of the pro- and mesotarsi and also on the metabasitarsus; (iii) predatory **3** (i) tarsi reduced to three tarsomeres; (ii) loss of the fastigium verticis; (iii) development of stridulatory file on ventral surface of tegminal CuP; (iv) presence of a dividing vein and harp between CuA2 and CuP **4** (i) tegminal medial fan expanded, forming a subapical medial lobe **5** (i) prothoracic legs fossorial; (ii) ovipositor vestigial **6** (i) compound eyes markedly reduced; (ii) all coxae large and closely approximated; (iii) pseudosegmented cerci; (iv) reduced ovipositor; (v) obligate inquilines of ants **7** (i) development of a tegminal mirror **8** (i) migration of dividing veins in the mirror to a position perpendicular with respect to long axis of tegmen.

Figure 4 shows a tentative reconstruction of relationships among major ensiferan groups based on a review of hypotheses presented in the various studies summarized in Fig. 3. The phylogenetic relationships of the gryllidean taxa are based primarily on the molecular phylogeny of Legendre et al. (2010; refer to Fig. 3i herein) with minor modification regarding the uncertain position of the obscure and highly derived Myrmecophilidae. We mapped phylogenetically informative morphological characters onto this topology and lists of character transformations are provided for each node in the figure caption. As discussed above, there appears to be considerable congruence between morphological and molecular data. Moreover, our attempt to place fossil Grylloidea (Protogryllidae and Baissogryllidae) on this phylogeny did not radically alter the topology. Indeed, whilst the monophyly of these taxa is questionable, their relationships are clearly delimited in the current hypothesis. In particular, the Protogryllidae almost certainly represent a paraphyletic grade of basal crickets that gave rise to all other Grylloidea sometime between the Late Triassic and Late Jurassic. The oldest protogryllids are known from the mid-Triassic (Carnian, *c*. 225 Ma) Molteno Formation of South Africa (Gorochov and Rasnitsyn 2002) which would suggest that the schizodactylid lineage is at least mid-Triassic in age. Such an extensive ‘ghost lineage’ is not uncommon among orthopterans, which are often frustratingly rare as fossils.

Whilst it is clear that major group relationships within Ensifera remain largely unresolved (see Legendre et al. 2010), the morphological and molecular support for a Schizodactylidae–Grylloidea sister-group relationship is compelling. Moreover, evidence for a close relationship between Schizodactylidae and Gryllacrididae or any of the other ‘stenopelmatoid’ families (Zeuner 1939; Sharov 1968; Gorochov 1995a, b, 2001) is based either on misinterpretation of non-homologous structures as homologous autapomorphies (e.g. gryllacridid pulvilli and the lateral processes of schizodactylid tarsi) or characters known to be plesiomorphic in Ensifera (e.g. four-segmented tarsi, ventral subapical spurs on the metatibiae etc.). A sister-group relationship between Grylloidea and the Triassic Gryllavidae as proposed by Gorochov (1995a, b) is difficult to demonstrate given that the latter family are known only from fossil wings. However, the tegminal venation of Gryllavidae is similar to that of Cyrtophyllitinae and it is likely that Gryllavus and related genera are closer to the paraphyletic “hagloid” assemblage than to the Grylloidea. With this in mind, and given the lack of convincing morphological or molecular autapomorphies for Stenopelmatoidea *sensu* Gorochov (2001), it seems reasonable to accept a schizodactylid–grylloid clade as shown in Fig. 4 until evidence is presented to the contrary.

## Supplementary Material

XML Treatment for Schizodactylidae

XML Treatment for Schizodactylus

XML Treatment for Schizodactylus groeningae
